# Supplementary material for: Hot-Carrier Generation in Bimetallic Janus Nanoparticles
Source: ACS Nano. 2026 Jan 29;20(5):4291–300. doi: 10.1021/acsnano.5c17401 (PMC12895562; doi:10.1021/acsnano.5c17401)
Supplement: Supplementary file 1 [file nn5c17401_si_001.pdf]

Supplementary information for ‘Hot-carrier generation in bimetallic Janus nanoparticles’

Hanwen Jin,[†,‡] Chengcheng Xiao,[†] Matias Herran,[¶] Emiliano Cortés,[¶] Shiwu Gao,[‡] and Johannes Lischner[*,†,§]

†*Department of Materials, Imperial College London, England*

‡*Material and Energy Division, Beijing Computational Science Research Centre, China*

¶*Nanoinstitut München, Fakultät für Physik, Ludwig-Maximilians-Universität München, Munich, Germany*

§*The Thomas Young Centre for Theory and Simulation of Materials, London E1 4NS, United Kingdom*

E-mail: j.lischner@imperial.ac.uk

Tight-binding model

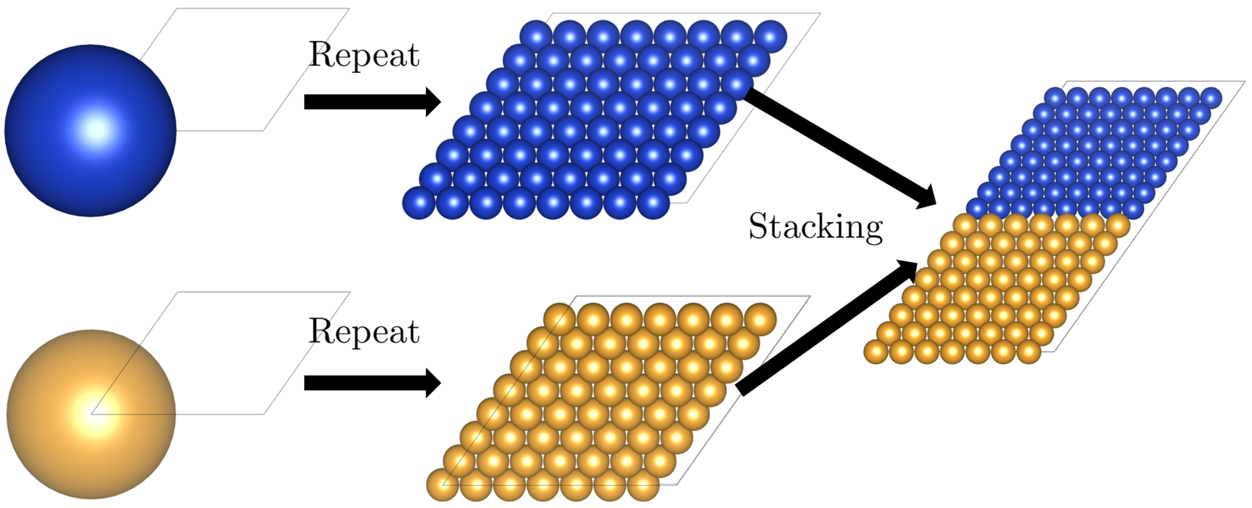

Figure S1: Schematic illustration of the construction of the bimetallic interface used for fitting tight-binding models for Janus nanoparticles.

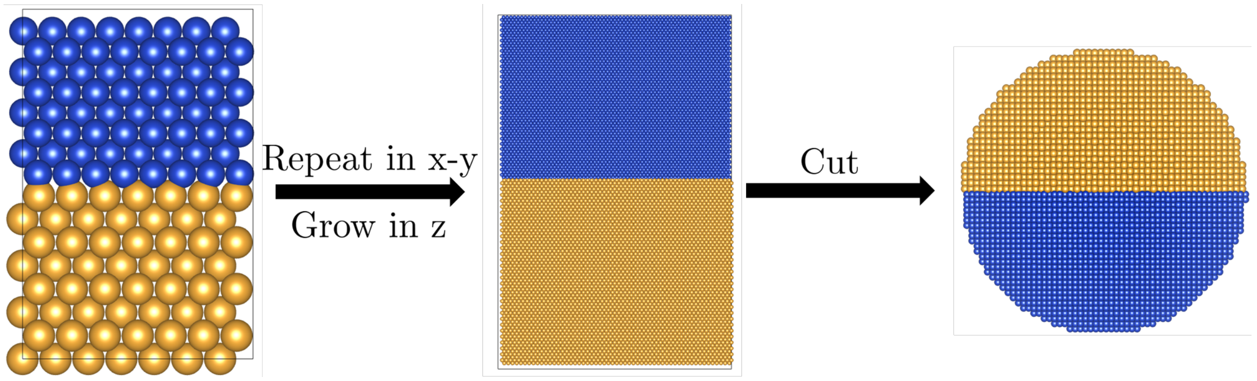

Figure S2: Schematic illustration of construction of a Janus nanoparticle from the atomistic interface model.

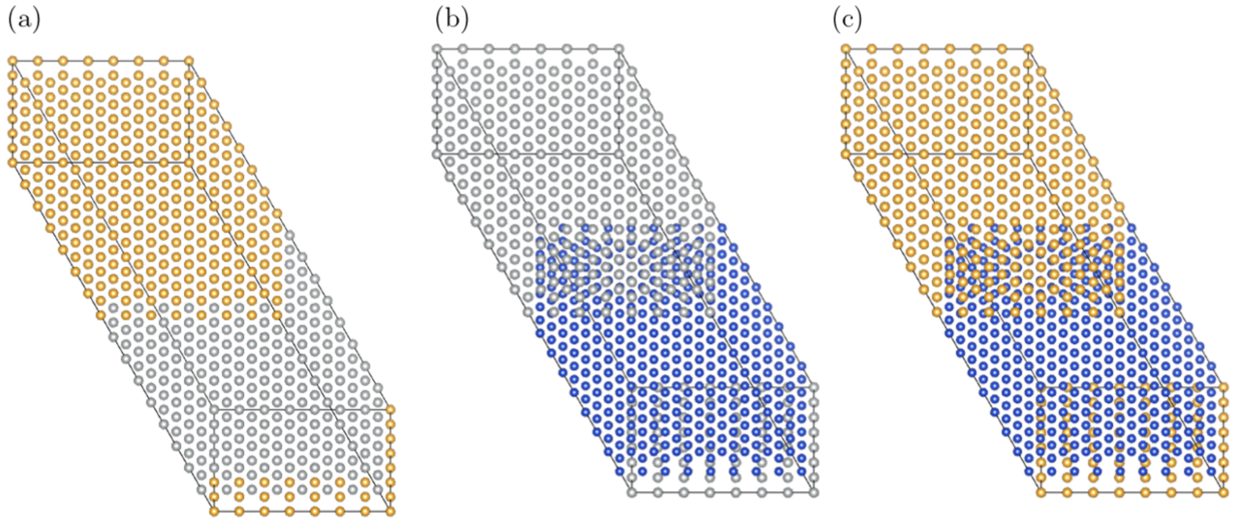

Figure S3: Atomic structure of the supercells used in the DFT calculations of the bimetallic interfaces. (a) Ag-Au interface, (b) Ag-Cu interface, (c) Au-Cu interface. Silver atoms are coloured in silver, gold atoms in gold and copper atoms in blue.

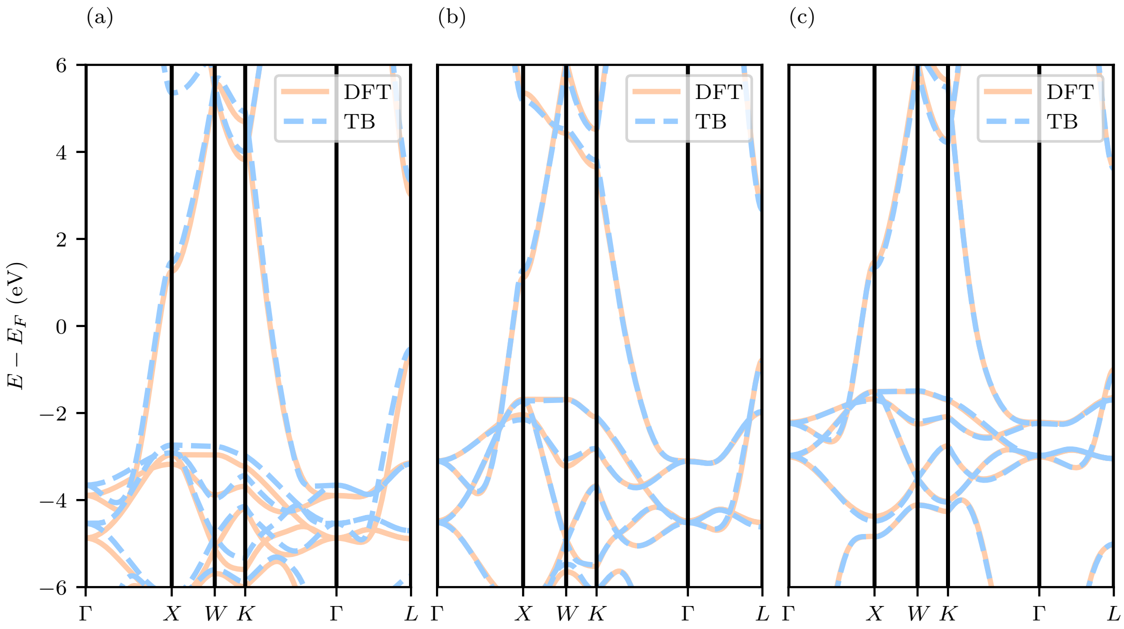

Figure S4: Bulk band structures of (a) Ag (b) Au (c) Cu calculated from DFT and tight binding.

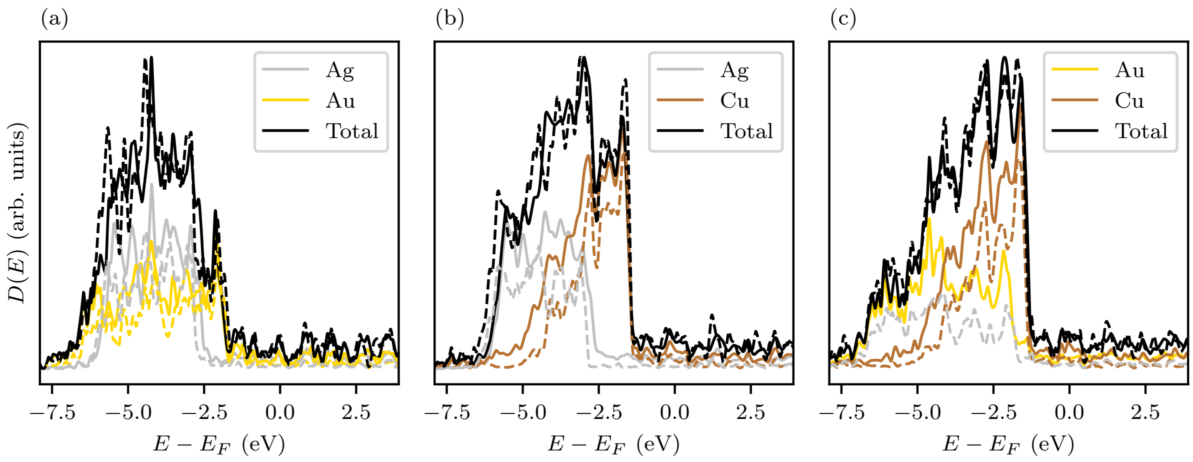

Figure S5: Density of states of (a) the Au/Ag interface, (b) the Cu/Ag interface, (c) the Cu/Au interface from tight-binding (solid lines) and the density functional theory (dashed lines). We have set the zero of the x-axis to the Fermi level of the system.

Table S1: The Slater-Koster parameters (in eV) of Ag, Au and Cu. $\Delta E_{\mathrm{Ag/Au/Cu}}$ denote the onsite energy shifts which are added to all onsite energies to ensure an accurate energy level alignment when a bimetallic interface is considered.

	Ag	Au	Cu
$ss\sigma1$	-0.82304	-0.88415	-1.03045
$pp\sigma1$	2.10945	2.05236	2.25499
$pp\pi1$	-0.05893	0.01567	0.07597
$dd\sigma1$	-0.41541	-0.63442	-0.35511
$dd\pi1$	0.26730	0.28405	0.23146
$dd\delta1$	-0.06275	-0.02080	-0.04289
$sp\sigma1$	1.17440	1.31924	1.45484
$sd\sigma1$	0.04737	-0.65608	-0.48483
$pd\sigma1$	-0.03112	-0.92976	-0.56336
$pd\pi1$	0.49614	0.18467	0.19503
$ss\sigma2$	-0.10597	0.03318	0.03886
$pp\sigma2$	0.19297	0.44646	0.58136
$pp\pi2$	0.12400	0.05051	0.05634
$dd\sigma2$	-0.04779	-0.03163	-0.05987
$dd\pi2$	0.04316	0.00435	0.02339
$dd\delta2$	0.00738	-0.00639	-0.00577
$sp\sigma2$	0.01595	-0.06003	0.08758
$sd\sigma2$	0.51901	-0.09838	-0.13260
$pd\sigma2$	0.04368	-0.17655	-0.11278
$pd\pi2$	0.32017	0.00579	0.01895
E_s	10.52798	9.55722	10.17554
E_p	16.86127	17.14594	16.43749
E_d	3.59990	5.09343	4.75432
a_0	4.15000	4.15000	3.63125
ΔE_{Ag}	0.0000	0.60000	-0.10000
ΔE_{Au}	-0.55000	-0.00000	-0.70000
ΔE_{Cu}	0.2000	0.80000	0.00000

Additional hot carrier figures

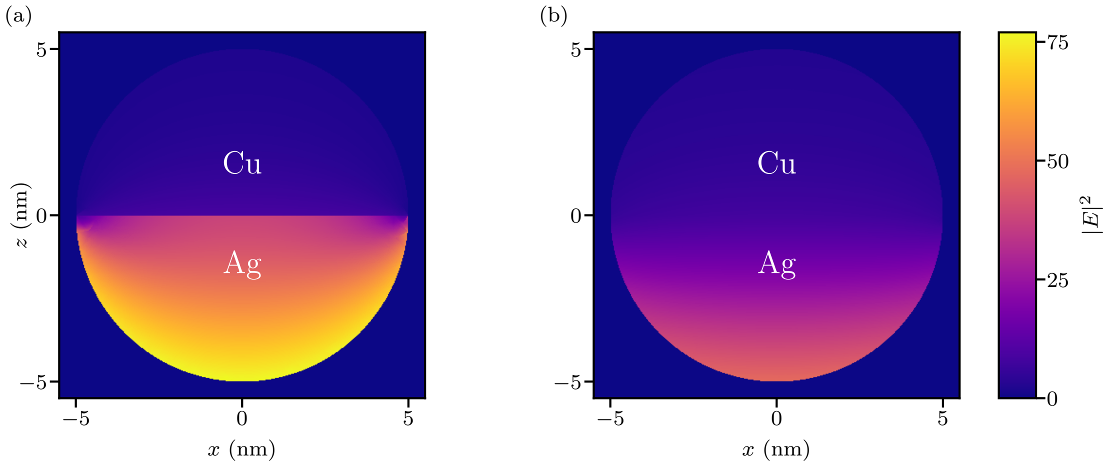

Figure S6: (a) The electric field intensity profile for spherical Ag-Cu nanoparticle at $\theta = 0°$ and $\hbar\omega = 3.4$ eV. (b) The electric field intensity profile for Ag-Cu at $\theta = 90°$ and $\hbar\omega = 3.4$ eV.

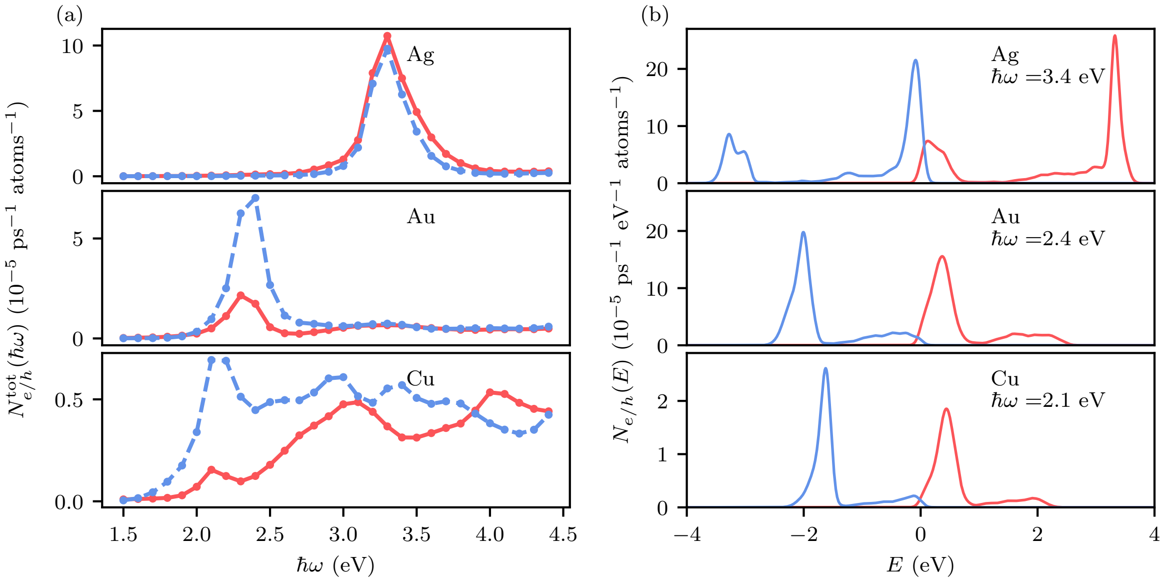

Figure S7: Hot-carrier generation in spherical mono-metallic nanoparticles of Ag, Au and Cu. (a) Generation rates of highly energetic holes (blue curves) and electrons (red curves), (b) energy-resolved hot-carrier rate at the respective plasmon frequencies. The radius of all nanoparticles is 10 nm. The illumination intensity is set to 1 mW μm^{-2}.

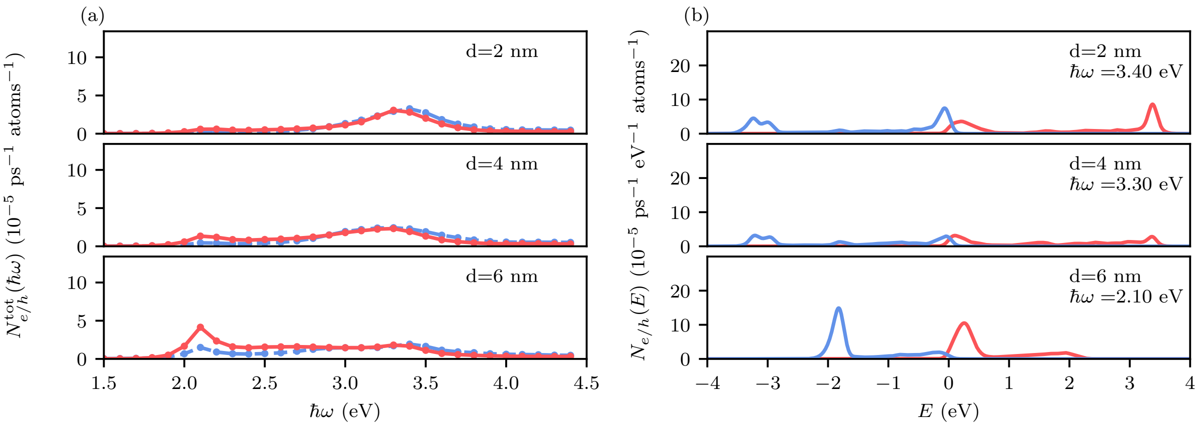

Figure S8: (a): Total generation rate of highly energetic electrons (red solid line) and holes (blue dashed line) as function of photon energy for Ag-Cu Janus nanoparticles with different neck sizes. Highly energetic carriers are defined as having energies larger than 1 eV relative to the Fermi level. (b): Energetic distribution of hot electrons (red lines) and hot holes (blue lines) in Ag-Cu Janus nanoparticles with different neck sizes at the lower-energy localized plasmon resonance frequencies. All energies are relative to the Fermi level. We used polarisation at $\theta = 0^o$.

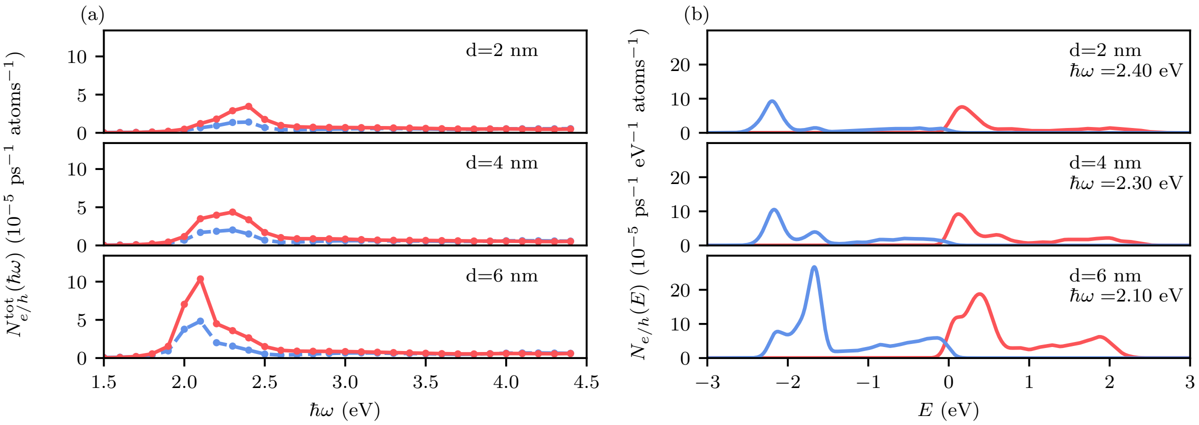

Figure S9: (a): Total generation rate of highly energetic electrons (red solid line) and holes (blue dashed line) as function of photon energy for Au-Cu Janus nanoparticles with different neck sizes. Highly energetic carriers are defined as having energies larger than 1 eV relative to the Fermi level. (b): Energetic distribution of hot electrons (red lines) and hot holes (blue lines) in Au-Cu Janus nanoparticles with different neck sizes at the lower-energy localized plasmon resonance frequencies. All energies are relative to the Fermi level. We used polarisation at $\theta = 0^o$.

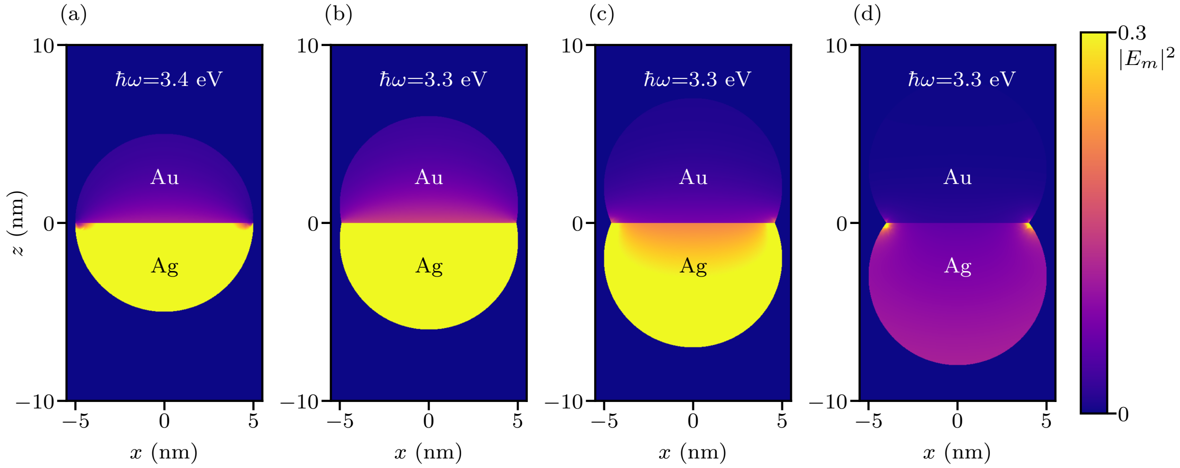

Figure S10: Electric field squared $|\mathbf{E}|^2$ (in unit of E_0) inside the four dumbell shaped Ag-Au nanoparticle considered in this thesis for the Ag plasmon (a) $d = 0$ nm, with maximum intensity of 77, (b) $d = 2$ nm, with maximum intensity of 73, (c) $d = 4$ nm, with maximum intensity of 129 and (d) $d = 6$ nm, with maximum intensity of 364, the polarisation is set at $\theta = 0$. For better visualization purpose, have set the maximum value of in the color plot to be 0.3 times the absolute maximum value.

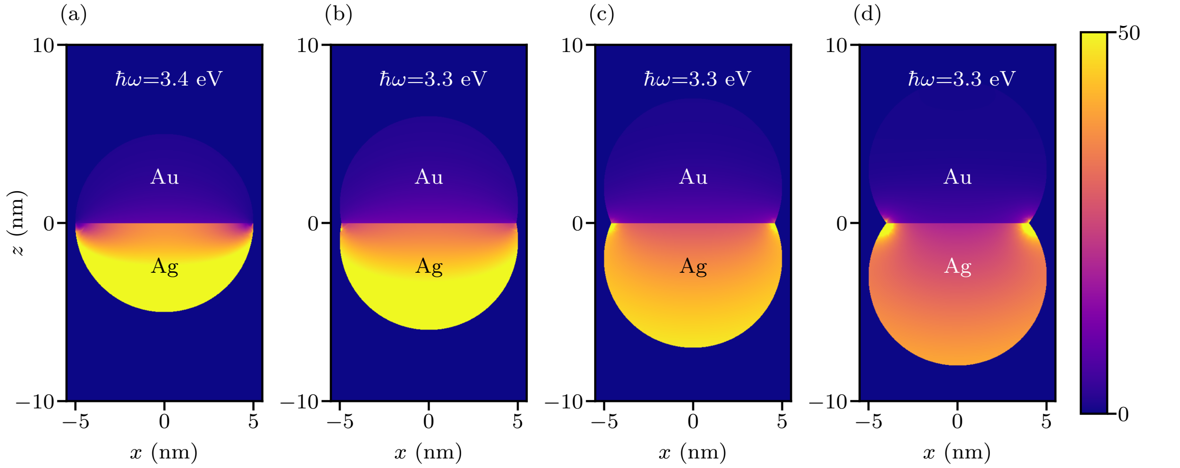

Figure S11: Electric field squared $|\mathbf{E}|^2$ (in units of $|E_0|^2$) at the Ag plasmon energy inside dumbbell-shaped Ag-Au Janus nanoparticles with (a) $d = 0$ nm, (b) $d = 2$ nm, (c) $d = 4$ nm, and (d) $d = 6$ nm. The polarization is set to $\theta = 0$. To better visualize the field in the Ag nanoparticle, we set the maximum square electric field in all panels to 50.

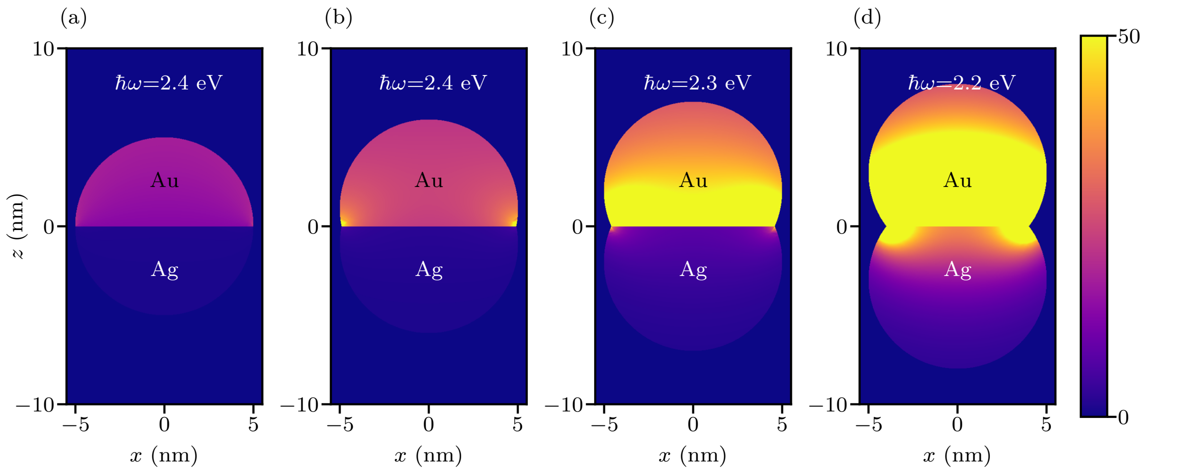

Figure S12: Squared magnitude of the electric field $|\mathbf{E}|^2$ (in units of $|E_0|^2$) at the Au plasmon energy inside dumbbell-shaped Ag-Au nanoparticles with neck sizes (a) $d = 0$ nm, (b) $d = 2$ nm, (c) $d = 4$ nm and (d) $d = 6$ nm. The electric field is perpendicular to the interface ($\theta = 0°$). To better visualize the field in the body of Au nanoparticle, we set the maximum squared electric field of all panels to 50.

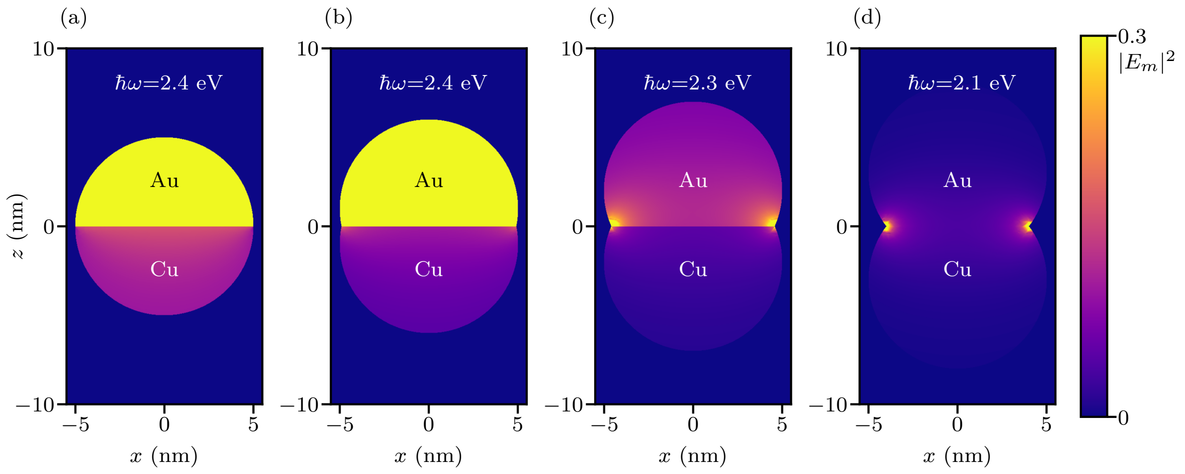

Figure S13: Electric field squared $|\mathbf{E}|^2$ (in unit of E_0) inside the four dumbell shaped Au-Cu nanoparticle considered in this thesis (a) $d = 0$ nm, with maximum intensity of 15.7, (b) $d = 2$ nm, with maximum intensity of 36.9, (c) $d = 4$ nm, with maximum intensity of 191 and (d) $d = 6$ nm, with maximum intensity of 1630, the polarisation is set at $\theta = 0$. For better visualization purpose, have set the maximum value of in the color plot to be 0.3 times the absolute maximum value.

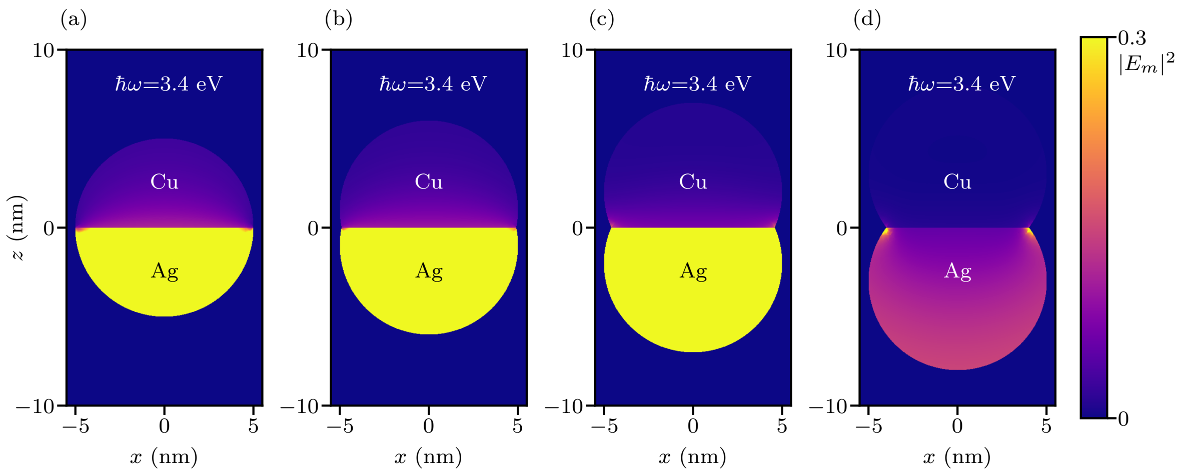

Figure S14: Electric field squared $|\mathbf{E}|^2$ (in unit of E_0) inside the four dumbell shaped Ag-Cu nanoparticle considered in this thesis (a) $d = 0$ nm, with maximum intensity of 78, (b) $d = 2$ nm, with maximum intensity of 75, (c) $d = 4$ nm, with maximum intensity of 73 and (d) $d = 6$ nm, with maximum intensity of 245, the polarisation is set at $\theta = 0$. For better visualization purpose, have set the maximum value of in the color plot to be 0.3 times the absolute maximum value.

Hot-carrier generation and absorbed power

According to macroscopic electrodynamics, the power density absorbed at position $\mathbf{R}$ inside the nanoparticle is given by

$$P(\mathbf{R}, \omega) = \frac{1}{2}\epsilon_2(\omega)\omega|\mathbf{E}(\mathbf{R}, \omega)|^2, \tag{S1}$$

where ϵ_2 is the imaginary part of dielectric constant.[1]

To established the relationship between absorbed power and hot-carrier generation, we first consider a uniform electric field with potential $\Phi(\omega) = -E(\omega)\hat{z}$ acting on the electronic system. The hot-electron generation rate per unit volume (in atomic units) can be written as

$$N_e(E, \omega) = \frac{4\pi|E(\omega)|^2}{V}\sum_{ia}|\langle i|\hat{z}|a\rangle|^2\delta(E_a - E_i - \omega)\delta(E - E_a)f_i(1 - f_a). \tag{S2}$$

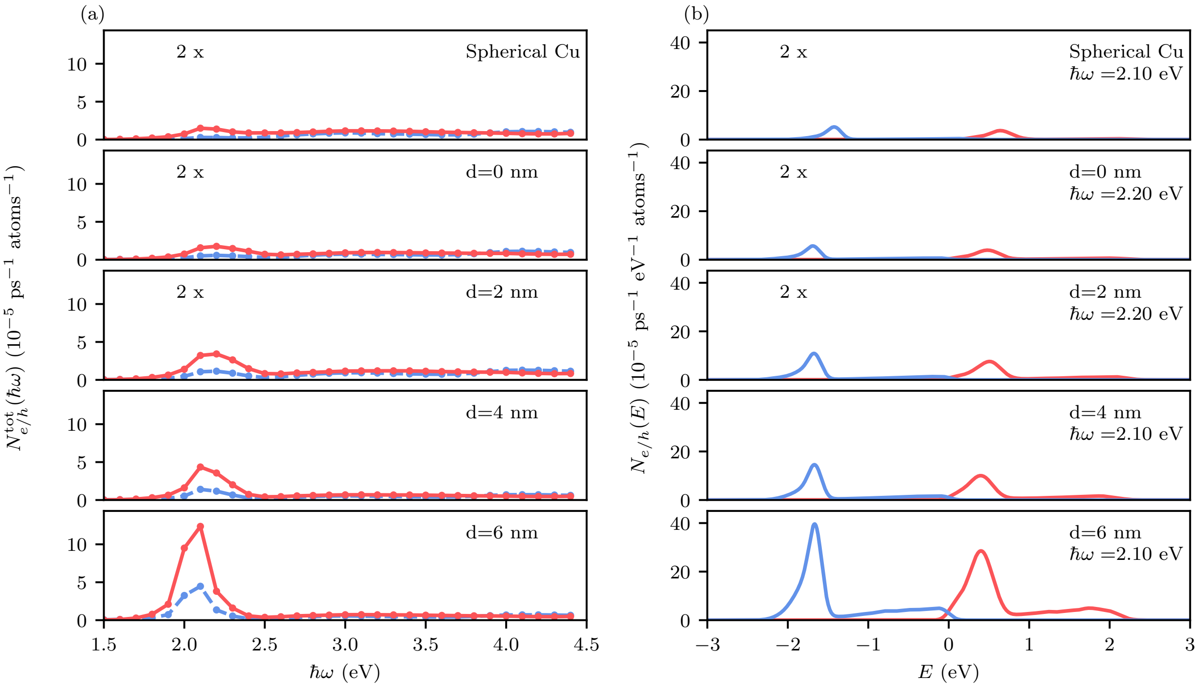

Figure S15: (a): Total generation rate of highly energetic electrons (red solid line) and holes (blue dashed line) as function of photon energy for the Copper part of Au-Cu Janus nanoparticles with different neck sizes, the top panel corresponds to spherical Cu nanoparticle as a reference. Highly energetic carriers are defined as having energies larger than 1 eV relative to the Fermi level. We separated the Cu contribution by treating Au as a dielectric, which is present in COMSOL multiphysics® but absent in tight-binding. (b): Energetic distribution of hot electrons (red lines) and hot holes (blue lines) in Cu of Au-Cu Janus nanoparticles with different neck sizes at the lower-energy localized plasmon resonance frequencies. All energies are relative to the Fermi level. We used polarisation at $\theta = 0^{\rm o}$.

The imaginary part of the dielectric function is given by[2,3]

$$\epsilon_2(\omega) = \frac{8\pi}{V} \sum_{ia} |\langle i|\hat{z}|a\rangle|^2 \delta(E_a - E_i - \omega) f_i (1 - f_a). \tag{S3}$$

As each electron-hole pair carries energy ω (and the total number of electron-hole pairs is equal to the total number of excited electrons), the total absorbed power is

$$V\omega \int N_e(E,\omega) E dE = \frac{V}{2}\omega |E(\omega)|^2 \epsilon_2(\omega) = \int P(\mathbf{R},\omega) d\mathbf{R}. \tag{S4}$$

This result shows that the energy-integrated hot-carrier generation rate is directly proportional to the power density, which in turn is proportional to $|\mathbf{E}|^2$.

For spatially varying electric fields, it is not possible in general to directly establish a proportionality between the local hot-carrier generation rate and $|\mathbf{E}(\mathbf{R})|^2$. However, if the electric field varies slowly in space, we can locally expand the potential in a Taylor series according to

$$\Phi(\mathbf{R}) \approx \Phi(\mathbf{R}_0) - \mathbf{E}(\mathbf{R}_0,\omega) \cdot (\mathbf{R} - \mathbf{R}_0). \tag{S5}$$

If we further assume that the electronic structure in the vicinity of $\mathbf{R}$ is bulk-like (this is certainly not appropriate in the vicinity of the bimetallic interface), we can use Eq. S3 to express the local hot-electron generation rate in a similar form as Muravitskaya et al..[1] We have also compared the absorbed power obtained from a solution of Maxwell's equation (within the quasistatic approximation) and from our approach which combines a solution of Maxwell's equation with a large-scale tight-binding simulation, see figure S16. Both methods are found to be in good agreement with each other.

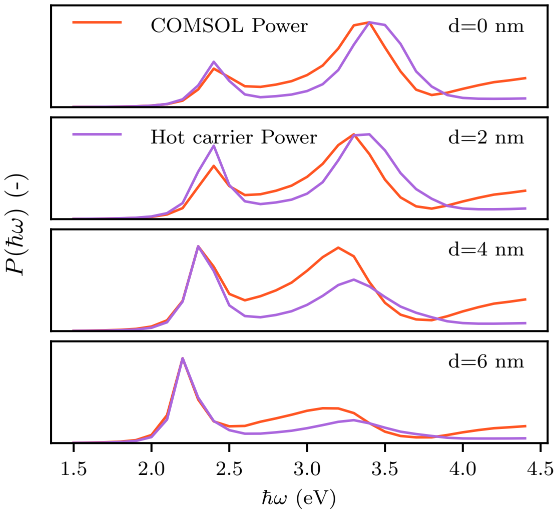

Figure S16: Absorbed power as function of photon energy for a Ag-Au Janus nanoparticle with different neck sizes obtained from the quasistatic approximation (labeled “COMSOL Power”) and from our approach which combines a solution of the Maxwell equation with a large-scale tight-binding simulation (labeled “Hot carrier Power”). For easier comparison the curves have been rescaled so that they have the same maximum value.

References

(1) Muravitskaya, A.; Movsesyan, A.; Ávalos-Ovando, O.; Bahamondes Lorca, V. A.; Correa-Duarte, M. A.; Besteiro, L. V.; Liedl, T.; Yu, P.; Wang, Z.; Markovich, G.; Govorov, A. O. Hot Electrons and Electromagnetic Effects in the Broadband Au, Ag, and Ag–Au Nanocrystals: The UV, visible, and NIR Plasmons. *ACS Photonics* **2024**, *11*, 68–84.

(2) He, Y.; Zeng, T. First-Principles Study and Model of Dielectric Functions of Silver Nanoparticles. *The Journal of Physical Chemistry C* **2010**, *114*, 18023–18030.

(3) Wang, L.-W.; Zunger, A. Dielectric Constants of Silicon Quantum Dots. *Physical Review Letters* **1994**, *73*, 1039–1042.
